# Primary oral mucosa-associated lymphoid tissue (MALT) lymphoma in patient with monoclonale gammopathy: a rare case report

**DOI:** 10.1186/s12903-021-01960-y

**Published:** 2021-11-23

**Authors:** Hilal Hafian, Hubert Schvartz, Martine Patey, Anne Quinquenel

**Affiliations:** 1 Département Médecine et Chirurgie Orales, Faculté d’Odontologie, Université de Reims Champagne Ardenne, 2, Rue du Général Koenig, 51100 Reims, France; 2 Service de Chirurgie Orale, Centre Hospitalier Universitaire de Reims, Hôpital Maison Blanche, 45, Rue Cognac Jay, 51100 Reims, France; 3 Laboratoire de Recherche en Nanosciences (LRN), EA 4682, Université de Reims Champagne Ardenne, Reims, France; 4 Service de Pathologie, Hôpital Robert Debré, CHU de Reims, Reims, France; 5 Expert Centre of Anatomopathological Network LYMPHOPATH, CHU de Reims - Hôpital Robert Debré, Reims, France; 6 Service d’Hématologie Clinique, Hôpital Robert Debré, CHU de Reims, Reims, France; 7 Réunion de Concertation Pluridisciplinaire Hématologie, Hôpital Robert Debré, CHU de Reims, Reims, France; 8 Département de Médecine et Chirurgie Orales, Pôle de Médecine Bucco-Dentaire, Centre Hospitalier Universitaire de Reims, 45, Rue Cognacq-Jay, 51100 Reims, France

**Keywords:** Mucosa-associated lymphoid tissue (MALT), Monoclonal gammopathy, Lymphoma, Oral mucosa, Light chain, Haemopathy, Head and neck, Extra nodal

## Abstract

**Background:**

Monoclonal gammopathy is a biological reality encountered in approximately 1% of the general population. In the absence of clinical and biological signs, it is considered of undetermined significance; however, it can be a biological signature of a monoclonal lymphocytic or plasma-cell proliferation. Their localisation to the oral mucosa remains rare and difficult to diagnose, particularly in indolent forms that escape imaging techniques.

**Case presentation:**

Here, we report the case of a 73-year-old woman with a history of IgM kappa gammopathy followed for 13 years. The patient did not have a chronic infection or an autoimmune disease, and all the biological investigations and radiological explorations were unremarkable during this period. The discovery of a submucosal nodule in the cheek led to the diagnosis of MALT lymphoma and regression of half of the IgM kappa level after resection. The review of the literature shows the dominance of clinical signs (i.e., a mass or swelling) in the diagnosis of primary MALT lymphomas of the oral cavity after surgical resection.

**Conclusions:**

Our case illustrates the role of examination of the oral cavity in the context of a monoclonal gammopathy. The absence of clinical and radiological evidence in favor of lymphoplasmacytic proliferation, does not exclude a primary indolent MALT lymphoma of the oral mucosa.

## Background

Monoclonal gammopathy of undetermined significance (MGUS) is defined as serum-M protein lower than 30 g/L, fewer than 10% clonal plasma cells (PCs) in the bone marrow (BM) and, most importantly, the absence of organ damage that can be attributed to PC proliferative disorder [1]. It is an asymptomatic condition characterised by the absence of any isolated monoclonal lymphocytic and/or plasmocytic proliferative component forming a tumour and the absence of any other biological abnormality. This is the case for more than 60% of monoclonal gammopathies [2]. MGUS is present in 1–3% of the general population over 50 years of age and up to 5% of those over 70 years of age [3]. They are mostly fortuitous discoveries made in the course of a systematic medical check-up motived by various symptoms or biological abnormalities, which are at first glance unrelated to a malignant haematolymphoid disorder. However, monoclonal gammopathy can also be discovered due to a complication related to the monoclonal component, primarily autoimmune, such as cytopenia, coagulopathy, autoimmunity or cryoglobulin activity. The renal complications associated with MGUS have been identified as a new entity called monoclonal gammopathy of renal significance (MGRS) [4]. The main complications of MGUS are the risk of progression to malignant haemopathy, such as multiple myeloma (MM) (isotype IgG, IgA and light chains without heavy chains) or B-cell lymphoproliferative haemopathy (mainly IgM isotype) [5]. The risk of progression is approximately 1% per year [6]. but almost 90% of MGUS cases will never develop lymphoproliferative malignancies. This is why the current clinical recommendation is long-term clinical monitoring for the majority of patients with MGUS [1]. A monoclonal gammopathy, however, can be a blood biological signature of secretory PCs in lymphoplasmacytic disorders, as in MM, extramedullary or solitary plasmacytoma, primary amyloidosis and Waldenström macroglobulinaemia, but also in approximately one-third of mucosa-associated lymphoid tissue (MALT) lymphomas [7] or other B-lymphoproliferative disorders. MALT lymphomas, which are subtypes of non-Hodgkin lymphomas, are B-cell lymphomas of the extranodal marginal zone B cells [8, 9]. They constitute approximately 11% of B-cell lymphomas associated with lymphoid tissues of the mucosa [10]. Most cases occur in adults, in the sixth and seventh decades, with slight predominance for females [9]. These lesions are characterised by their tendency to remain localised and are often set in an inflammatory background due to infection or autoimmunity. The most common site for these lymphomas in the gastrointestinal tract is the stomach, where they are mostly related to a *Helicobacter pylori* infection; however, MALT lymphomas can occur in other common sites, including salivary glands, lung, head and neck mucosa, ocular adnexa, skin, thyroid and breast [11–13]. The risk of development of MALT lymphoma is increased in patients with autoimmune diseases such as Sjögren’s syndrome, Hashimoto’s thyroiditis or lymphoepithelial sialadenitis.

MALT lymphomas may also occur in the oral cavity; this location is extremely rare and is most frequently discovered by clinical manifestations such as swelling or a submucosal mass [10, 14]. We report the case of primary indolent MALT lymphoma localised in the cheek, discovered as an infra-centimetric submucosal mass in a 73-year-old woman who had been followed for more than 13 years for an IgM kappa MGUS that decreased after surgical excision of the nodule of the cheek.

## Case presentation

**Fig. 1:**
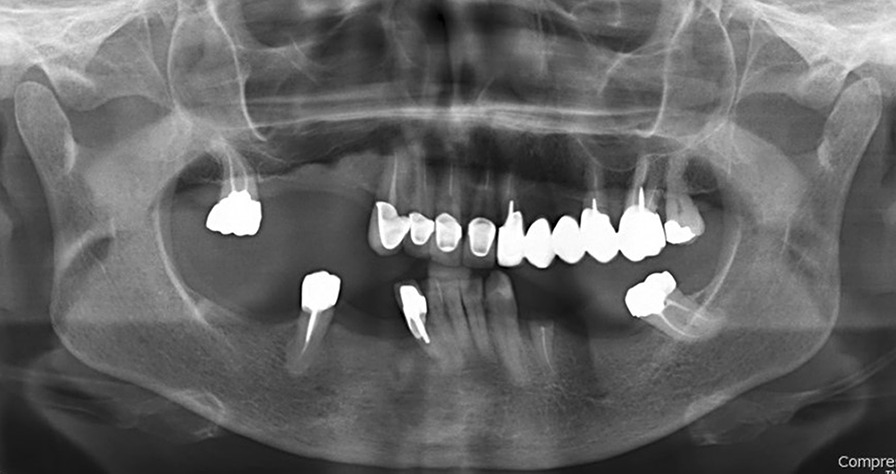
Panoramic radiography at the first consultation negative for pathological bone imaging

A 73-year-old female was referred by a dentist for the extraction of the residual root of the second right mandibular premolar (Fig. 1). She was followed for 13 years for an IgM kappa monoclonal gammopathy with a serum level at 13.16 g/L at the time of consultation. The patient had no autoimmune disease history. Medical imaging during the 13-year follow-up of the gammopathy did not identify any lesions or suspicious images. All computed tomography (CT) and magnetic resonance imaging (MRI) of the pelvis, abdomen, chest and head and neck were without abnormality. A total body positron emission tomography/computed tomography (PET/CT) was performed routinely, since the diagnosis of monoclonal gammopathy did not show suspect hypermetabolic focus. Biological analysis of the patient’s blood count was without abnormality, C-reactive protein level increased gradually over the 13 years of follow-up, and all sternal BM aspirations performed were also without abnormality.

**Fig. 2:**
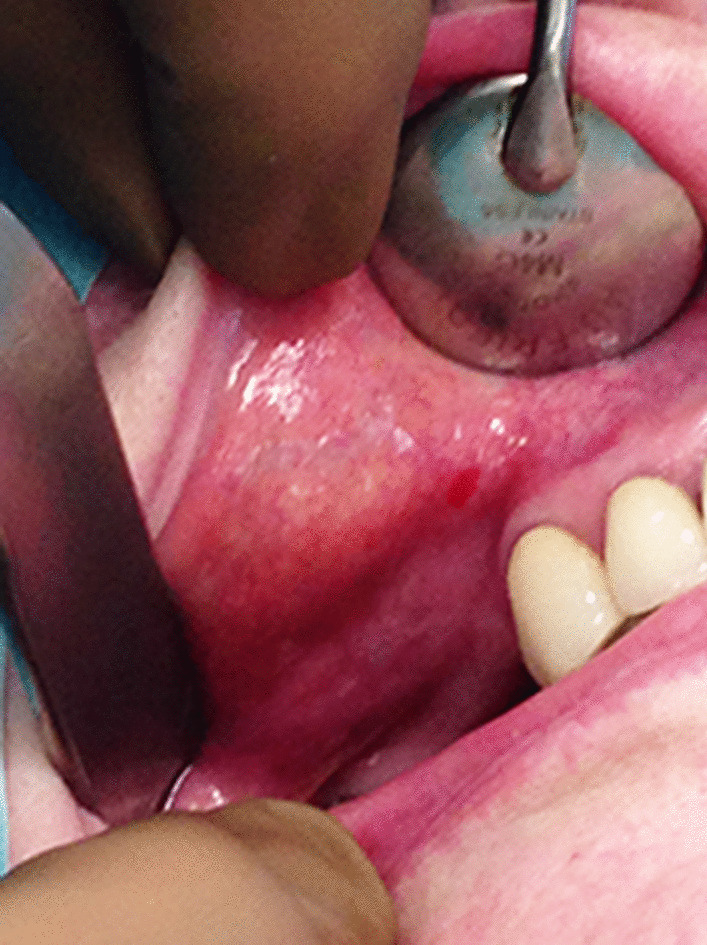
Intraoral view of submucosal nodule of the right check

**Fig. 3:**
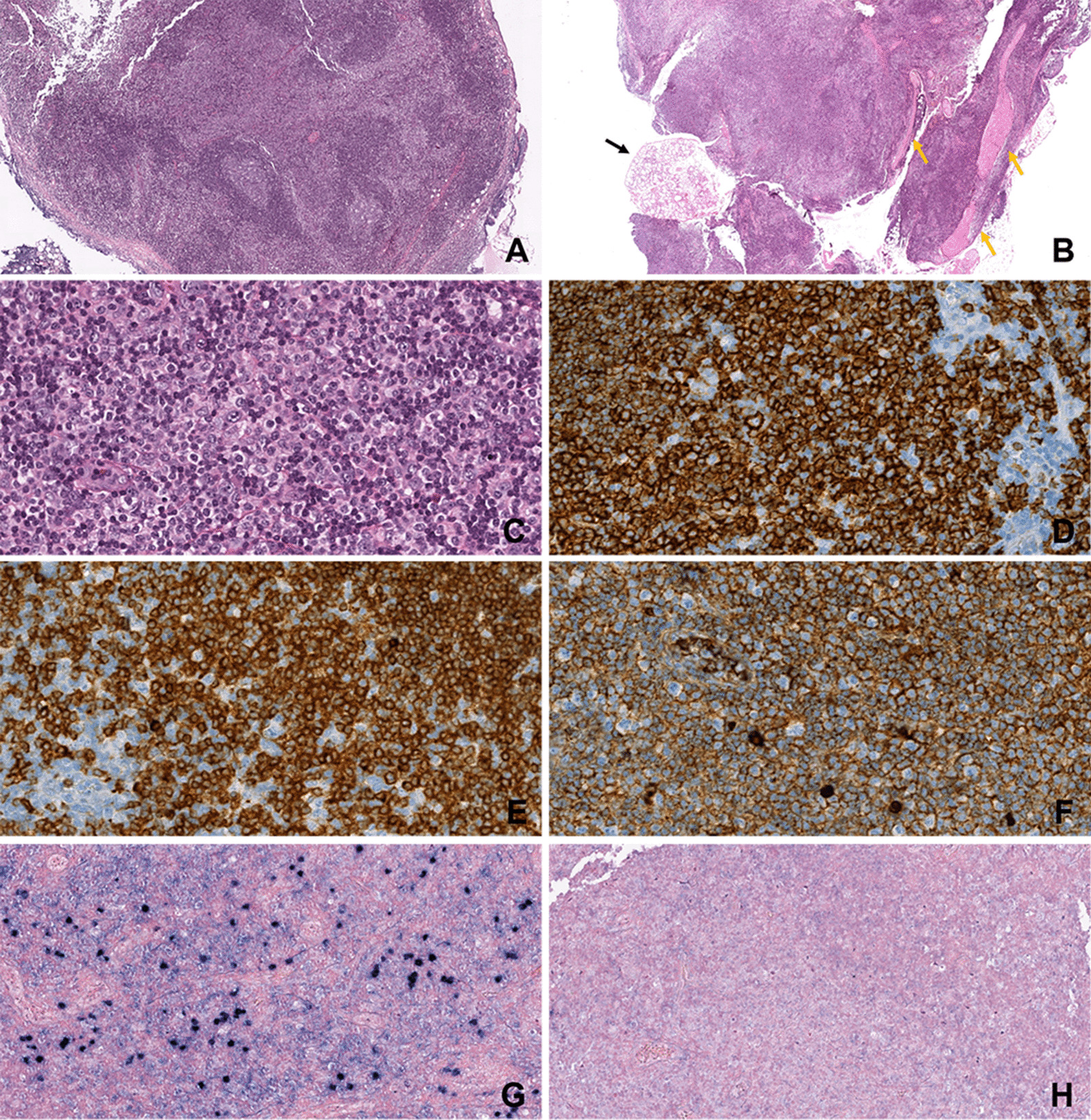
**A** and **B** Light examination of tumour, low magnification (haematoxylin and eosin stain, original magnification × 20), **A** Pseudo-nodular architecture of the excised lesion with some colonised lymphoid follicles with widened mantle zone in the MALT lymphoma, **B** Massive colonisation of the neoplastic follicles; tumour cells are scattered and infiltrate adjacent cellulo-adipose tissue with insheathing of the small nervous fillets (yellow arrow), but do not invade the healthy lobule of salivary gland (black arrow). No epithelial or salivary structure is observed in the tumour infiltrate, **C** Tumour cells of small size with an abundant and pale cytoplasm and irregular nuclei; these cells have rather dense chromatin nuclei with a cytoplasm sometimes off-centred and a plasma-cell differentiation (original magnification × 40). **D**, **E** Immunohistochemical staining (original magnification × 40), **D** Strong positive for CD20, **E** Strong positive for CD79a. **F** Uniform strong staining of membrane cells for IgM (original magnification × 20). **G**, **H** In situ hybridisation for light chain of immunoglobulin (original magnification × 40) **G** High expression of kappa light chain **H** Absence of the expression of lambda light chain

The intraoral visual examination did not show any mucosal lesions; however, salivation was normal and the patient had no symptoms, especially pain or swelling. A thorough systematic physical examination was performed, with free bidigital palpation of the floor of the mouth and left cheek, but palpation of the right cheek revealed a submucosal infra-centimetric and irregular nodular lesion underneath the orifice of the Stenon canal (Fig. 2). It was not fixed to the deep plane and was of firm consistency. No cervical lymphadenopathy was found. In the context of monoclonal gammopathy, surgical excision of this nodule was performed under local anaesthesia, at the same operating time as the extraction of the residual root of the second right mandibular premolar. The excised tissue consisted of a brownish yellow tissue measuring 0.8 cm × 0.7 cm × 0.5 cm. On pathological examination, the lesion was firm and the cut surface yellowish-white. Microscopically, dense lymphomatous proliferation of the pseudo-nodular architecture was present, revealing some germinal centres colonised by a lymphomatous infiltrate, which also widened the marginal zone and infiltrated the interfollicular areas (Fig. 3a). Proliferation of the tumour tended to massively infiltrate the germinal centres with invasion of the adipose tissue and insheathing of the nerve filaments (Fig. 3b). The diffuse proliferation of tumour cells in the marginal zone was formed of cells of small size, with irregular nuclei, but that were rarely nucleated. The tumour cells had rather dense chromatin nuclei with a cytoplasm that was sometimes off-centred and a plasma-cell differentiation (Fig. 3c), We found some mast cells. There were no Dütcher body-type intra-nuclear inclusions. Immunohistochemical study of the lymphomatous proliferation showed that the tumour cells expressed CD20 and CD79a (Fig. 3d, e), monoclonal heavy chain IgM (Fig. 3f), and kappa light chain (Fig. 3g) and were negative for lambda light chain (Fig. 3 h).

Many tumour cells expressed CD5- and anti-CD3-staining marked T-lymphocytes. A network of cells was immunolabelled for CD10, BCL2, BCL6, MUM1 and cyclin D1. The BCL2 and MUM1 markers were expressed by many PCs. CD21 and CD23 highlighted the dendritic cells in residual dislocated germinal centres. Histological examination did not show lymphoepithelial structure, and the anti-AE1/AE3 immunolabelling was negative. The final diagnosis was primary MALT lymphoma.

The patient was referred to the Haematology department, and no indication of specific treatment was retained in the absence of symptoms or high tumour burden. With a follow-up of 4 years, no local recurrence or dissemination was observed clinically or in annual CT scans. Biological assessment, particularly thyroid, hepatic and renal, remained normal, and electrophoresis of the plasma proteins showed a decrease in the serum level of IgM kappa to 7 g/L, probably favoured by the surgical excision of the primary isolated MALT lymphoma of the mucosa of the cheek.

## Discussion

MGUS is present in roughly 3–4% of the population over 50 years of age and in approximately 5% of those older than 70 years [3] and virtually precedes the development of MM and related disorders, i.e., lympho-plasma-cellular neoplasms, Waldenström macroglobulinaemia, light-chain amyloidosis and also non-Hodgkin’s lymphoma (NHL) [15]. It has been defined by the International Myeloma Working Group (IMWG) as a plasma-cell disorder characterised by a serum monoclonal level lower than 30 g/L, BM PCs lower than 10% and absence of organ damage, lytic bone lesions, hypercalcaemia or renal failure, and which can be attributed to a plasma-cell proliferative disorder [1, 16, 17]. The IgM MGUS is a MGUS subtype that presents a risk of progression to NHL, and especially Waldenström macroglobulinaemia [18], whereas non-IgM MGUS (i.e., IgA, IgG or, rarely, IgD or IgE) are assumed to be associated with the risk of plasma-cell tumour development, although definitive diagnosis should include BM examination [19, 20], Prediction of the risk progression of MGUS patients who will remain stable compared to those who progress is very difficult at the time of recognition of the MGUS; however, the level and type of M protein, the number of BM PCs (if a BM aspiration is performed) and the free light-chain ratio, can be used to stratify the risk of progression of MGUS to MM or related disorders [21] and enable monitoring and patient management as dictated by the IMWG guidelines [22].

Non-Hodgkin’s lymphomas of the oral cavity are rare tumours. They represent 10–15% of extranodal lymphomas and less than 5% of malignant tumours of the oral cavity. This is the third most common tumour group after squamous cell carcinoma and malignant tumours of the salivary glands [23, 24]. B-cell lymphoma is the most common histological form, and over 70% of cases are of diffuse large B-cell (DLBCL)-subtype lymphomas with preferential localisation to the oropharynx, followed by follicular lymphoma (11.1%), and extranodal marginal zone MALT lymphoma (9.2%) [10].

MALT lymphoma is considered an extranodal variant of marginal zone B-cell lymphoma and is a distinct entity of NHL [9]. It is more frequent in women older than 60 years of age, but some cases have been reported in children, as shown by the literature data summarised in Table 1. This subtype of B-cell lymphoma has a better prognosis than its nodal counterparts, with a rather indolent evolution and a tendency to remain localised, as approximately only a quarter of the cases tend to disseminate to multiple sites. The most common site of development is the stomach, and the majority of gastric MALT lymphomas are associated with *Helicobacter pylori* infection [25]. Salivary and thyroid MALT lymphomas are associated with autoimmune disorders, such as Sjögren syndrome and Hashimoto disease, respectively [26]. Their occurrence on the mucosa of the oral cavity as primary tumours is extremely rare [24, 27], and they present mainly as mass swellings on the tongue or as nodular forms in the lip or buccal mucosa (Table 1). The clinical manifestation may lead to the histological diagnosis on a biopsy sample or a resected specimen. In our reported case, the patient did not present any clinical manifestations or symptomatology. The nodule under the mucosal lesion was discovered fortuitously during a careful clinical examination of a 73-year-old woman with a history of IgM kappa MGUS for more than 13 years with no known or identified lesions and no specific clinical manifestation. No superficial adenomegaly or splenomegaly was highlighted on physical examination, but electrophoresis of plasma proteins showed an IgM level of 5.4 g/L at the time of MGUS discovery, which increased gradually to 13.16 g/L at the time of detection of the right cheek mucosal nodule. The LDH level was normal, and beta-2 microglobulin level was 2.45 mg/L. Blood tests for autoimmunity were performed (anti-Sm, anti-RNP, anti-SS-A, anti-SS-B, anti-Sc70, anti-JO-1) and found to be normal, as were thyrostimulin (TSH) and thyroxin (T4) levels. Renal and liver function tests, as well as blood cell count, were normal. No proteinuria was evidenced. Serology for C hepatitis (HCV) was negative, and endoscopic examination of the gastrointestinal tract showed no particularity. Overall, our patient presented no specific risk factors for the development of MALT lymphoma, more particularly, of the oral mucosa, which is a rare and atypical extra nodal localisation of this type of lymphoma.

**Table 1:** Clinical and pathological characteristics of reported cases of isolated oral MALT lymphoma without autoimmune or infection diseases

Case	References	Country	Age (years)	Sex	Site	CD5	Light chain		Clinical manifestation	Involvement MSG	Management	Recurrence
							Κ	λ				
1	Iftikhar et al. [40]	Pakistan	61	M	BoT	+	NK	NK	Dysphagia	−	CT	−
2	Song et al. [41]	Korea	29	F	BoT	NK	NK	NK	Mass involving T lymphoma history	−	RT	−
3	Gotri et al. [42]	Italy	80	F	BoT	+	+	−	Dysphagia	−	SR	−
4	Ferry et al. [33]	USA	62	F	BoT	+	−	+	Mass	−	NK	Spread
5	Kojima et al. [14]	Japan	51	M	SP	−	+	−	ND	+	SR	−
6			60	M	BM	−	−	−	ND	−	SR + RT	−
7			64	F	SP	−	+	−	ND	+	SR	+
8			78	F	Ging	−	−	−	ND	−	RT	−
9			83	M	Ging	−	−	−	ND	−	CT	+
10	Sakabe et al. [43]	Japan	61	F	BoT	+	NK	NK	Mass	+	SR	−
11	Ayers et al. [44]	USA	64	F	HP	NK	NK	NK	Mass	+	NK	NK
12	Bombeccari et al. [45]	Italy	11	M	LL	−	+	NK	Swelling	−	SR	−
13	Kojima et al. [46]	Japan	78	F	BM	−	−	+	Swelling	−	NK	NK
14	Kolokotronis et al. [47]	Greece	68	M	Ts	NK	NK	NK	NK	NK	SR + CT	+
15			73	F	HP	NK	NK	NK	NK	NK	SR	+
16			48	F	Ts	NK	NK	NK	NK	NK	SR	+
17	Crandley et al. [48]	USA	9	F	LL	NK	−	+	Swelling	+	SR	−
18	Frazier et al. [49]	USA	50	M	UL	−	+	−	Swelling	+	SR	−
19	Abe et al. [50]	Japan	64	F	HP	+	+	+	Swelling	−	SR + RT + CT	NK
20	Berrebi et al. [51]	France	10	M	LL	NK	NK	NK	Mass	+	SR	−
21	Eder [52]	UK	52	M	FoM	NK	NK	NK	Mass	NK	SR	−
22	Gabali et al. [53]	USA	11	M	LL	−	−	−	Swelling	+	SR	NK
23	Ruy et al. [54]	Korea	7	F	LL	−	+	−	Mass		CT	−
24	Tanaka et al. [35, 36]	Japan	66	F	BM	+	+	+	Swelling	−	SR	−
25	Gerami [55]	USA	57	F	LL, To then UL	−	NK	NK	Swelling	−	CT	+
26	Honda et al. [56]	Japan	71	F	FOM	NK	+	NK	Swelling	NK	RT	−
27	Kaplan et al. [57]	Israel	59	F	LL	−	NK	NK	NK	+	CT + anti-CD20	−
			87	F	LL	−	NK	NK	Mass	+	SR	−
			82	F	UL	−	NK	NK	Mass	+	SR	−
			82	F	LL	−	NK	NK	Mass	+	SR	−
			66	F	LL	−	NK	NK	Mass	+	SR	−
			71	F	UL and later LL	−	NK	NK	Mass	−	SR	−
33	Kawasaki et al. [58]	Japan	27	M	UL and LL	NK	NK	NK	Mass	+	SR	−
34	Song et al. [59]	Korea	29	F	BoT	−	NK	NK	Mass		RT	−
35	Mo et al. [60]	USA	12	M	LL	−	+	−	Mass	+	CT	−
36	Zehani et al. [61]	Tunis	28	F	Cheek	−	+	−	Swelling	−	NK	NK
37	Zambrano et al. [62]	USA	14	M	UL	−	+	+	Mass	NK	NK	NK
38	Urano et al. [63]	Japan	62	F	Cheek	NK	−	+	Swelling		RT + anti-CD20	−
39	Ma et al. [64]	China	59	F	Ts	−	−	−	Swelling	−	SR + RT	−
40	Manveen et al. [65]	India	40	M	HP	+	NK	NK	Swelling	−	SR	−
41	Bianco et al. [66]	Italy	82	M	UL	−	NK	NK	Swelling	−	CT + RT	−
42	Tauber et al. [67]	Germany	71	F	HP	NK	NK	NK	Mass	NK	SR	−

*MSG* minor salivary gland, *BOT* base of the tongue, *SP* soft palate, *BM* buccal mucosa, *Ging* gingiva, *HP* hard palate, *LL* lower lip, *Ts* tonsil, *UL* upper lip, *FoM* floor of the mouth, *To* tongue, *SR* surgical resection, *CT* chemotherapy, *RT* radiotherapy, *NK* not known, *–/+* absence/presence of marker expression (CD5, Κ, λ), absence/presence of salivary gland structure, no recurrence/recurrence

MALT lymphomas of the oral cavity can be delineated into two distinct types: the first arises from a pathologic lymphocyte infiltrate of the minor salivary glands, and the second from lymphoid cells of the mucosa, independent of inflammatory salivary disease. Under normal circumstances, the parenchyma of the salivary glands do not contain lymphocyte infiltrate, but they can acquire it in inflammatory pathological conditions, especially in Sjögren syndrome [28], which is a systemic autoimmune pathology with a lymphoepithelial sialadenitis as a hallmark feature, but also in other chronic pathologies, such as sclerotic sialadenitis or localised inflammation [29, 30] In our patient, the minor salivary gland lobule observed on the examined histopathological slides appeared healthy and did not show any inflammatory infiltrate (Fig. 3b); moreover, no lymphoepithelial structure was identified on anti-AE1/AE3 immunolabelled slides of the tumour tissue. These features were in favour of a primary MALT lymphoma of the oral mucosa in an atypical localisation to the cheek mucosa.

The pathological features of MALT lymphoma of the head and neck region and therefore, in the oral cavity, are similar to those of MALT lymphoma occurring elsewhere. Its immunophenotypic profile shows that lymphoma cells express B lineage markers, such as CD20, with a variable proportion of plasmocytic differentiation expressing CD79a membrane protein in one-third of cases [31]. They usually express IgM and sometimes IgG or IgA, but not IgD. Monotypic immunoglobulin light-chain expression is essential for differential diagnosis with benign lymphoid infiltrates.

The expression of CD5 by MALT lymphomas is not frequent. Differential diagnoses of other forms of CD5-expressing small-cell lymphomas, such as mantle cell lymphomas and small lymphocytic lymphomas, should be made. The absence of expression of cyclin D1 distinguishes them from mantle cell lymphomas, and the absence of CD10 expression achieves differential diagnosis from most follicular lymphomas. MALT lymphomas are typically negative for CD5, CD10, BCL6 and cyclin D1. In a study of 14 cases of MALT lymphomas, Jaso et al. showed that CD5-positive immunolabelling seems to be associated with extragastric locations with a tendency to dissemination [23], but no cases were localised in the oral cavity in their series. However, CD5-positive oral MALT lymphomas have been associated with disseminated forms [32, 33], and several cases of MALT lymphomas of the oral mucosa expressing both kappa and lambda light chains have been correlated with aggressive behaviour and recurrence [8, 34, 35]. Tanaka et al. reported a case of a primary MALT lymphoma of the oral mucosa that was CD5-positive and expressed kappa light chain at the time of the diagnosis, and then expressed both light-chain lambda and kappa in the recurrent lesion [35–37].

The association between the phenotypic expression profile of CD5 and the lambda and kappa light chains in MALT with either an aggressive or recurrent character remains unclear, and the CD5 phenotype can be expressed in indolent form [35], as in our patient who had a monoclonal gammopathy for more than 13 years without any lesions or suspicious radiological images. Given the age and evolution of our patient’s MALT lymphoma, it can be considered indolent, although at the time of diagnosis the tumour cells were CD5-positive, in addition to kappa light chain expression. The absence of local recurrence or dissemination over 4 years of follow-up and, more specifically, the marked reduction of the kappa light chain monoclonal peak by electrophoresis of plasma proteins 4 years after surgical excision, reinforces the hypothesis of a primary and indolent MALT lymphoma of the oral mucosa.

Active treatment options for the management of MALT lymphoma include surgery, radiotherapy, chemotherapy, immunotherapy and combined modalities. The optimal choice is based on the disease stage and primary anatomical site. The treatment sequence for primary MALT lymphoma is assimilated to the management of an extranodal marginal zone lymphoma, with, from our observation, a specificity for oral mucosa location and a late diagnosis of the tumour in a patient with no chronic infection or dysimmunity and with a long history of MGUS. In addition, the pathological diagnosis is made based on surgical biopsy with complete excision. The tumour can be considered an isolated, single site of primary extranodal, non-gastric MALT lymphoma and can be stratified at the stage I of Lugano staging system or with a more modern system, such as the T1m N0 M0 of Paris staging system [38] for gastrointestinal tract lymphoma. In the absence of a target infectious agent, a practical guideline [39] must take into account the site of involvement and the potential for organ dysfunction, whether it is localised or disseminated, the morbidity associated with any proposed treatment, and the general physical condition of the person. In our case, a single-site MALT lymphoma was localised at the oral mucosa of the cheek, which is a site that does not contain an organ with a potential risk of dysfunction. The patient was in very good physical condition, with a World Health Organization (WHO) index of 0. Under these conditions, a ‘watch and wait’ approach was considered, and observation without therapy was established by a multidisciplinary consultation meeting of haematology. Four years after surgical excision and lymphoma diagnosis, no local recurrence or spreading evolution were observed. This is in agreement with the observations of primary MALTs of the oral cavity reported in the literature (Table 1), in which 21 of the 42 cases (50%) were treated by surgical excision alone, with a predominant localisation at the lips (10/21), and 17 of the 21 cases (81%) showed no progression or recurrence during the follow-up period. Of note, all reported cases had a clinical manifestation as a first sign; in particular, swelling or a small mass. This partly explains surgical management with total excision for histopathological diagnosis. In our case, the small submucosal nodule was discovered during a thorough physical examination of the oral cavity in the context of MGUS followed for 13 years, which motivated surgical excision. The other cases reported in the literature (17/42) were treated by different associations of therapeutic modalities, combining surgery, radiotherapy, chemotherapy and anti-CD20 immunotherapy with variable results and poor and unhelpful information, given the very low number of cases.

## Conclusions

This reported case is, to our knowledge, the first diagnosis of a primary oral mucosa MALT lymphoma in a patient with a monoclonal gammopathy followed for a significant amount of time. It reminds all professionals of oral medicine and surgery of the importance of meticulous and systematic physical examination of the oral mucosa, which can be the site of expression of a systemic disease and, notably, of malignant haemopathy, with a weak clinical manifestation and non-specific, especially in indolent forms. This can modify the prognosis of progression of the monoclonal gammopathy, which represents a precancerous state with a risk of developing MM or lymphoproliferative haemopathy.

## Data Availability

Not applicable.

## Abbreviations

MALT: Mucosa-associated lymphoid tissue
MGUS: Monoclonal gammopathy of undetermined significance
PCs: Plasma cells
MGRS: Monoclonal Gammopathy of renal significance
MM: Multiple myeloma
CT: Computer tomography
PET/CT: Positron emission tomography/computer tomography
MRI: Magnetic resonance imaging
NHL: Non-Hodgkin’s lymphoma
IMWG: International Myeloma Work Group
DLBCL: Diffuse large B-cell lymphoma
TSH: Thyroid-stimulating hormone
T4: Thyroxin
HCV: C-Viral hepatitis
WHO: World Health Organization
H&E: Hematoxylin and eosin
